# Structural Conservation and Adaptation of the Bacterial Flagella Motor

**DOI:** 10.3390/biom10111492

**Published:** 2020-10-29

**Authors:** Brittany L. Carroll, Jun Liu

**Affiliations:** 1 Department of Microbial Pathogenesis, Yale School of Medicine, New Haven, CT 06536, USA; Brittany.Carroll@yale.edu; 2 Microbial Sciences Institute, Yale University, West Haven, CT 06516, USA

**Keywords:** bacterial flagellum, cryo-electron tomography, cryo-electron microscopy, molecular motor, structure and function, torque generation, evolution

## Abstract

Many bacteria require flagella for the ability to move, survive, and cause infection. The flagellum is a complex nanomachine that has evolved to increase the fitness of each bacterium to diverse environments. Over several decades, molecular, biochemical, and structural insights into the flagella have led to a comprehensive understanding of the structure and function of this fascinating nanomachine. Notably, X-ray crystallography, cryo-electron microscopy (cryo-EM), and cryo-electron tomography (cryo-ET) have elucidated the flagella and their components to unprecedented resolution, gleaning insights into their structural conservation and adaptation. In this review, we focus on recent structural studies that have led to a mechanistic understanding of flagellar assembly, function, and evolution.

## 1. Introduction

**Figure 1:**
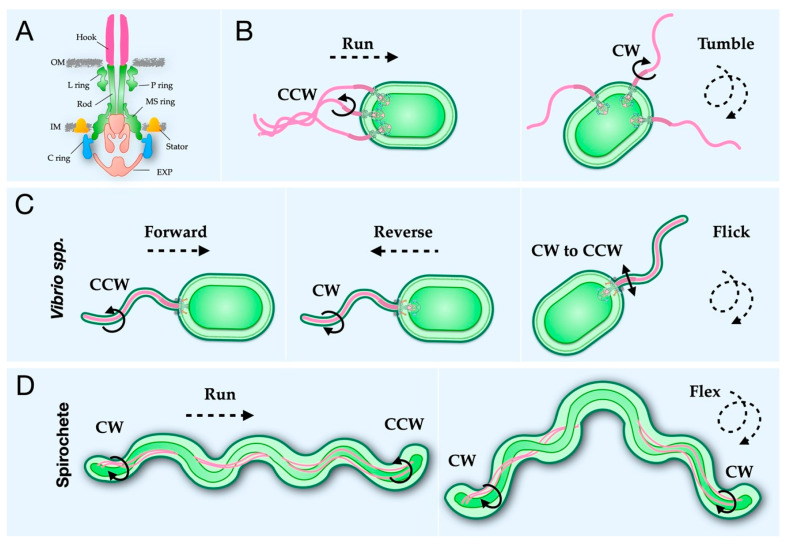
Bacterial flagella control distinct motility. The flagellar motor is a complex nanomachine that drives filament rotation. (**A**) Cartoon model of the flagellar motor. (**B**) In the two-step model used by many species, such as *E. coli* and *Salmonella*, the cell body is propelled forward, or runs, during counterclockwise (looking from the motor to the filament, CCW) rotation, and the filaments form an organized bundle. To change direction, the cell tumbles by rotating the filament in the clockwise (CW) direction, unwinding the bundle. (**C**) *Vibrio* spp. use a three-step method, with CCW rotation moving the cell body forward, CW rotation moving the cell body in reverse, and a flicking motion when CW-to-CCW randomly change direction. (**D**) Spirochetes, with periplasmic flagella at both poles, require a unique two-step method. During the run, the flagella rotate CCW and CW at opposite poles, such that one pole “pulls” while the other “pushes”. Both poles rotate in the CW direction while the cell tumbles to change direction.

The flagellum, a complex nanomachine, propels bacteria through media and along surfaces, using an ion gradient across the cytoplasmic membrane (for review [1]). All flagella share basic structural elements, including the filament, hook, and motor (Figure 1A). The filament acts as the propeller guiding the bacterium through space, while the hook acts as a joint transmitting energy from the motor to the filament [2,3,4,5,6]. The motor, or basal body is homologous to the non-flagellar type III secretion system (T3SS) (for review [7]). The filament can present either externally (Figure 1B,C) or periplasmically (Figure 1D). External flagella extend through the outer membrane into the media surrounding the bacterium and can further be categorized as lateral, peritrichous, and polar [8], while periplasmic flagella reside within the periplasmic space and are essential for spirochete motility [9]. 

The flagella of *Salmonella enterica* (henceforth called *Salmonella*) and *Escherichia coli* possess the best-studied motors, consisting of the membrane/supramembrane (MS) ring, cytoplasmic (C) ring, peptidoglycan (P) ring, lipopolysaccharide (L) ring, rod, stator, and export apparatus. The MS ring (FliF) acts a base upon which the motor sits, and the C ring (FliG, FliM, and FliN) controls the rotation sense [10,11,12,13,14,15,16]. The stator generates torque through ion gradients, mainly H^+^ (MotA and MotB) and sometimes Na^+^ (PomA and PomB), which drives the rotation of the C ring [14,15,17,18,19]. The rod (FlgB, FlgC, and FlgF, and FlgG) acts as a drive shaft, connecting the MS ring to the hook [20,21,22], and the L (FlgH) and P (FlgI) rings act as the bushings, providing support to the rotating rod [23]. The export gate complex, (FlhA, FlhB, FliP, FliQ, and FliR) and ATPase complex (FliH, FliI, and FliJ) [24,25,26] are responsible for the temporal and spatial assembly, ensuring that a functional flagellum is built [27]. Advances in structural biology techniques, specifically cryo-electron microscopy (cryo-EM) and cryo-electron tomography (cryo-ET), have led to the investigation of flagella from many other species, resulting in the identification of conserved and specifically adapted structural features. Cryo-ET uniquely allows for the visualization of flagellar structures in situ, without the necessity of isolation and purification of the complexes. In this review, we summarize the plethora of structural work that has widened our view of the assembly, adaptation, and evolution of bacterial flagella.

## 2. The Bacterial Flagellar Structure

Structural studies have illustrated how the flagellum is assembled and the unique features that have evolved in different species. X-ray crystallography is particularly powerful in unveiling many atomic structures of individual flagellar proteins as well as small subcomplexes (Table 1). These atomic models provide invaluable insight into the individual proteins and protein–protein interactions involved in flagellar assembly and aid in designing functional studies. Recently, cryo-EM has been increasingly utilized to provide both medium- and high-resolution structures of many flagellar subcomplexes, elucidating variable symmetry and complexity of the motor (Table 2). However, the flagellum as an intact organelle is far too complex and flexible for X-ray crystallography and cryo-EM. Cryo-ET coupled with subtomogram averaging [28] has the unique capacity to reveal the entirety of bacterial flagella in multiple species, depicting the relative arrangement of the rings and other protein complexes of the flagella in situ (Table 3). In this section, we review the structural information that not only is conserved but also provides a basis for understanding the functions.

### 2.1. The Rod, Hook, and Filament Extend from the Cell Body

The flagellar filament is comprised of 11 protofilaments, each with thousands of repeating units of flagellin (for review [129]). Although, variation of the filament is possible, such as in the case of *Campylobacter jejuni* with 7 protofilaments [93]. The flagellin protein (FliC) has four domains—D0, D1, D2, and D3 [30]—and the protofilaments can adopt both left- and right-handed helical rotations. The filament forms a left-handed supercoil when rotating CCW and a right-handed supercoil during CW rotation, together coined polymorphic switching [130,131,132]. The Namba group solved atomic models of locked right-handed and left-handed *Salmonella* filaments using cryo-EM, elucidating key interacting regions of the flagellin protein [94,95]. Recently, the *Bacillus subtilis* and *Pseudomonas aeruginosa* locked filaments were revealed by using cryo-EM as well [96]. Importantly, due to improved resolution, Wang et al. were able to predict point mutations involved in polymorphic switching, which will aid future work towards a better understanding of the filament rotation [96]. 

The hook, composed of ~120 copies of FlgE forming 11 protofilaments, has the critical job of joining the filament to the basal body, requiring a balance of rigidity and flexibility to allow the transfer of energy without breaking [133]. FlgE has 4 domains: D0 forms the channel, D1 forms the middle body, D2 forms the exposed surface, and Dc loops back in towards D0 [103]. Advances in cryo-EM enable high-resolution views of the hook as a bended structure during flagellar rotation [103,104] or the earlier structures that were limited to straight segments [45,94,99]. Different from the two-state model [134], these studies revealed 11 different subunit conformations, suggesting that each protofilament has unique interdomain interactions allowing for compression and extension as necessary during rotation. The super helical pitch of the hook is dependent upon the environment, with a helical pitch of 996 Å at pH 3.5 [103] and 1,290 Å at pH 8 [104], indicating that the environment also plays a role in the supercoiled form. 

The rod is the most proximal region of the axial structure and acts as the drive shaft. It can be divided into two regions: the proximal rod contains six monomers of FlgB, FlgC, and FlgF and nine monomers of FliE, and the distal rod contains 26 copies of FlgG [135,136,137]. Biochemical characterization of the rod proteins suggests that FliE associates with the MS ring [25,137] and also with the proximal protein assembly of FlgB, FlgF, and FlgC [138]. A cryo-ET study looking at flagellar assembly in the spirochete *Borrelia burgdorferi* broke down the assembly of the proximal rod, distal rod, hook, and filament using various deletion mutants, confirming the previous cellular studies [119]. A recent crystal structure of the core fragment of FlgG from *Salmonella* docked into the cryo-EM maps of the distal rod [139] and hook [102] identified the importance of the L-stretch in stabilization of the rod-hook junction [51]. There was also striking similarity between FlgG and FlgE, highlighting the fluidity of the rod-hook junction evolution [51]. Importantly, the static structures of the rod, hook, and filament observed by X-ray and cryo-EM lack the payload stress that occurs during flagellar rotation; therefore, different forces acting on these structures during filament rotation may alter their configuration.

### 2.2. The Periplasmic P and L Rings Stabilize the Rod

Analogous to the bushing, the P (FlgI) and L (FlgH) rings are located within the periplasmic space and encircle and stabilize the rod [23]. The L ring was thought to catalyze the removal of the rod cap protein, FlgJ [140]. Bioinformatic analysis suggests that the P and L rings are highly conserved yet evolved separately, rather than via horizontal gene transfer [141]. Visualization of the PL rings from a dozen diverse bacterial species further supports their conservation among phyla [141,142]. Recent cryo-ET studies have found that the P and L rings form the outer membrane structures when the flagellum is absent [125,142,143]. These novel structures, identified by different groups, have been called outer-membrane partial flagellar structures, flagellar outer membrane complexes (FOMCs), or PL subcomplexes [125,141,142,143]. These complexes were suggested to be relics from which flagella have detached or been sheared, as the rod appears to be required for the assembly of the subcomplexes. Furthermore, Zhu et al. did not observe FOMCs in a *P. aeruginosa flgG* mutant, suggesting that the distal rod is necessary for the formation of the FOMCs [143]. Interestingly, the sheathed flagellum (discussed below) of *Vibrio* spp. also possesses the PL subcomplexes [141], and spirochetes and firmicutes lack the L ring and PL ring, respectively [144], raising the question of whether there are still unknown functions of the P and L rings.

### 2.3. The MS Ring is the Base of the Motor

The MS ring, comprised solely of FliF, sits mainly in the periplasmic space but is anchored to the inner membrane via N- and C-terminal transmembrane helices [16,145]. FliF is a multidomain protein with two transmembrane domains, the ring-building motif domains (RBM) RBM1, RMB2, RBM3a, RBM3b, the β-collar domain, and C-terminal domain [146]. The C-terminal domain of FliF interacts with the N-terminus of the C-ring protein FliG [54,55], and the export gate complex resides within the MS ring [126,147]. A recent cryo-EM structural analysis of the MS ring answered the outstanding question of symmetry mismatch between the MS ring (25-fold) [106,108] and C ring (34-fold) [106,148,149]. The Lea group found that symmetry within the MS ring due to FliF folding creates an inner and outer ring. The export gate complex interacts with the 21/22-fold inner RBM domains, and the outer ring with 33/34-fold symmetry matches that of the C ring [146]. The unique organization of FliF allows the MS ring to grasp the rotor and export gate, acting to stabilize the basal body.

### 2.4. The C Ring Acts as a Rotor Within the Cytosol

The C ring, a notable structure located in the cytosol, is essential for flagellar rotation and assembly. The overall structure and shape are conserved, while the diameter of the C ring can vary across species [113]. Cryo-EM and cryo-ET studies have shown that *Salmonella* and *E. coli* have C rings with ~34-fold symmetry [148,150], and bacterial species with larger motors, such as ε-proteobacteria [114,121] and spirochetes, possess C rings with higher symmetry [116]. The increased resolution of cryo-ET has confirmed and expanded upon the initial observations of the C ring diameter variation.

Insights into the C ring composition were inferred from the homologous non-flagellar type III secretion system (or injectosome, for review [7]) of *Shigella* [69,151]. Using sequence alignments, mass spectroscopy, and cryo-EM, McDowell et al. suggested that multimerization of a repeating heptamer [151] containing FliG, FliM, and FliN creates a C ring with a spiral base in lieu of the previously postulated hexamer [152,153]. This finding has further been substantiated by bioinformatics techniques, establishing an evolutionary precedent [154] and pseudo-atomic models built into cryo-ET maps [120,128]. FliG, comprised of three domains (FliG_N_, FliG_M_, and FliG_C_), occupies the C ring adjacent to the MS ring and stator, with FliG_N_ interacting with FliF of the MS ring [54,55,155], and FliG_C_ interacting with MotA of the stator complex via charged residues [156,157,158]. FliM also contains three domains with similar nomenclature: FliM_N_ binds to CheY-P [67,159], FliM_M_ interacts with FliG_M_ [160,161,162], and FliM_C_ forms a heterodimer with FliN [69,154]. FliN is a single-domain protein that dimerizes with FliM or itself [163,164]. Numerous crystal structures of the C-ring proteins provide critical information on protein–protein interactions (Table 1). 

Some species have FliY, a protein with strong sequence homology to FliN and weak homology to FliM [163]. Typically, FliY replaces FliN, but in *Leptospira* and ε-proteobacteria, both FliY and FliN are expressed and necessary for flagellation [63,165]. The crystal structure of the FliN and FliY complex showed that these proteins form a heterodimer [63]. Co-expression and purification showed that *Campylobacter jejuni* FliY interacts with both FliN and FliM, but interestingly, FliN and FliM do not interact in ε-proteobacteria, *Helicobacter pylori*, or *C. jejuni* [63,166,167]. Recently, a detailed study of the *C. jejuni* C ring composition established the distinct roles of FliY and FliN, as they appear to have evolved independently. The FliY and FliM interactions are important for stabilization of FliH, while FliN is necessary for stabilization of the C ring, suggesting that the C ring is composed of a FliG–FliM–FliN–FliY complex in *C. jejuni* [122]. Understanding C ring composition has proven very important in revealing the switching mechanism for controlling the rotational sense (discussed below).

### 2.5. Torque is Generated by the Stator Through Ion Gradients

The stator complex generates the torque required to rotate the C ring through a proton gradient, although some species use Na^+^ ions [5,19,168,169]. Two membrane proteins, MotA and MotB, form the stator complex as the H^+^ powered pump, while the Na^+^-driven pump assembles from PomA and PomB [170]. The complexity of the stator complex is two-fold: (1) the stator complex undergoes conformational changes to gain functionality, and (2) the stator complex pool is known to be dynamic [171], leading to variations in stator assembly [172,173,174]. The dynamic nature of the stator complex makes trapping it with the motor during purification difficult. For these reasons, much of our knowledge of the conformational changes during stator assembly has been accumulated through biochemical experiments, although structural information is starting to accumulate [175]. 

Initially, cryo-EM structures of PomA/PomB and MotA uncovered the shape and organization of a stator subunit but lacked vital information about stator stoichiometry and rotor–stator interactions [109,171,176]. Freeze-fractured micrographs [177,178] and low-resolution cryo-ET [113,117,121,127,179,180] studies show the stator as a stud-like particle, with different species utilizing varying numbers of stators. Two recent high-resolution cryo-EM structures show that MotA:MotB and PomA:PomB exist in a 5:2 ratio [110,111]. Interestingly, one of these cryo-EM studies found very little conformational rearrangement of the stator complex during protonation in *C. jejuni*, using a protonation mimic mutant [110]. A cryo-ET study on *B. burgdorferi* greatly extended the resolution of the stator–C ring complex in situ, as the spirochete-specific collar of *B. burgdorferi* appears to stabilize the stator complexes around the C ring [116]. Mutations in MotB (D24N and D24E) result in non-motile and motile deficient spirochetes, respectively [116]. Furthermore, these mutations alter the number of stators assembled around the C ring; from these variations in stator number, C ring deformation increases with increased torque [116]. Cryo-ET partially resolved the elusive stator of *Vibrio alginolyticus* such that PomB appears to interact with MotX and MotY of the T ring, supporting the idea that the H and T ring help recruit the stators and allow for increased torque [181]. Evidently, bacteria have evolved sophisticated mechanisms to recruit stator complexes, perhaps to control torque necessary for different bacterial motility and behavior.

### 2.6. A Conserved Mechanism for Flagellar Rotational Switching

Bacteria move forward when the external flagella rotate in the CCW direction and tumble during CW rotation (Figure 1B) [5,56,160,169,182]. Notably, *Vibrio spp.* have a three-stroke swimming pattern, moving forward during CCW rotation, backward during CW rotation, and using a flicking motion upon CW-to-CCW rotation, analogous to the tumble (Figure 1C) [183]. Spirochete’s periplasmic flagellar rotation is unique as forward movement occurs when flagella at one pole rotate CCW and the other CW, and tumbling occurs when flagella at both poles rotate in the CW direction (Figure 1D) [120,184,185,186]. The C ring controls the rotational sense in response to chemical attractions and repellents [12]. A chemotaxis system mediates the rotational sense via cooperative binding of phosphorylated CheY (CheY-P) to FliM, resulting in a CCW motor switching to the CW sense [67,159] (for review see [187]). A co-crystal structure of CheY-P bound to a truncation of FliM_N_ provided direct evidence of this interaction [72]. The presence of CheY at the C ring has further been confirmed by two recent cryo-ET studies showing GFP-CheY at the outer periphery of the C ring. The first study used GFP-tagged CleD and CheY homolog in *Caulobacter cresecentus* [123], and the second used GFP-tagged CheY in *B. burgdorferi* [120]. 

The molecular mechanism of the C ring rotational switching has been extensively studied. High-resolution microscopy of fluorescently tagged FliM and FliN provided evidence of a high turnover rate of FliM and a slower but significant turnover of FliN [188,189,190,191]. Fluorescent studies of FliM suggest ~34 copies are in CW rotating motors and ~44 copies in CCW rotating motors [189]. It is still unknown what makes FliM appear more stable during CCW rotation. Cryo-EM studies of purified motors do not show the large change suggested by high-resolution light microscopy studies [191] but suggest a slight diameter difference between CCW and CW motors [192]. Two recent cryo-ET studies in *B. burgdorferi* and *V. alginolyticus* revealed the C ring conformational changes during rotational switching in situ [120,128]. These studies suggest that FliG–FliM–FliN stoichiometry remains consistent at 1:1:3 during switching, whereas there is a conformational change of the C ring subunits that leads to the different presentation of FliG to the stator. The stator complexes were resolved in the *B. burgdorferi* motor structure, showing direct evidence for a difference in FliG–MotA interactions between the two rotational senses [120]. Using cryo-EM coupled with functional assays, Santiveri et al. suggest that MotA of the stator unit in *C. jejuni* rotates, specifically in a clockwise direction during protonation [110]. Together, these studies support a new model for the C ring rotational switching, whereby the stator complex rotates in a CW manner, and the differences in the presentation of FliG to the stator complexes change the rotational sense of the C ring [110,111,120,128]. 

### 2.7. The Export Apparatus Secretes Flagellar Proteins for Assembly

The export apparatus is responsible for secreting proteins out of the cytoplasm and across the bacterial membranes to form a functional flagellum. Both proton motive force and ATP are utilized to unfold and translocate proteins across the cytoplasmic membrane. The export apparatus is composed of nine proteins: FlhA, FlhB, FliO, FliP, FliQ, FliR, FliH, FliI, and FliJ [24,25,118]. FlhA forms an ion channel [193,194,195,196] and has been shown biochemically and genetically to interact with multiple flagella-associated proteins [24,197,198,199]. FlhB, critical for substrate specificity, regulates the hook length and switching to flagellin secretion for filament assembly via an autocleavage event [200,201]. FliPQR forms the core complex, which is the channel that secretes the proteins [202]. The ATPase complex is formed by the ATPase (FliI), stalk protein (FliJ), and negative regulator (FliH) [7,118].

The first hints of structural and spatial information about the export apparatus came from freeze fracture experiments, establishing the presence of a pore [177,203]. Multiple cryo-ET studies proposed the location of the export apparatus [113,127,179,204]; however, Chen et al. were the first to study the structural detail export apparatus in depth [113]. By comparing flagella from many species, they showed that the export apparatus is highly conserved in shape and location, with a dome feature below the MS ring, a torus, and a spherical structure. A FliI deletion in *C. jejuni* resulted in intact flagella missing the spherical density, solidifying the location of the export apparatus, specifically the ATPase portion [113]. A recent cryo-ET study showed that the ATPase portion of the export apparatus is connected to the C ring via interactions with FliH and likely rotates with the C ring [118]. FliH is a negative regulator of FliI, but exactly how the assembled FliH–FliI complex is regulated is still unknown; the crystal structure, while revealing an intriguing FliH dimer, did not bare the assembled ATPase complex structure [92]. Deletion of *fliH* in *C. jejuni* led to loss of FliI density but still allowed for flagella assembly, providing direct evidence that the ATPase is non-essential for flagella assembly, consistent with biochemical results [122]. 

A proton channel in FlhA has been shown to be critical for powering the export of flagellar proteins [205,206,207,208]. FlhA is the largest protein of the export gate and contains three cytoplasmic domains, CD1 with the FHIPEP motif, a linker domain FlhA_L_ and a C-terminal domain FlhA_C_, as well as two transmembrane regions [193,209]. The C-terminal domain, which interacts with the chaperones and export substrate, has been crystallized and studied extensively but lacks structural information for the remaining regions [84,85,86]. Inferences of the FlhA structure can be drawn from a cryo-ET study of the *Salmonella* non-flagellar T3SS, in which a seahorse-shaped structure was resolved for InvA, the homolog to FlhA [210]. The FliPQR–FlhB complex has recently been resolved in multiple cryo-EM studies, whereby purified FliPQR and FliPQR–FlhB of the export gate complex from *Salmonella* revealed an unexpected topology and orientation of the complex, with no canonical transmembrane regions but rather with a helical structure that sits at the base of the basal body, mainly inside the periplasm [107,112,202]. These studies also confirmed, using native mass spectrometry, that both the flagellar and non-flagellar export gates have a P_5_Q_4_R_1_ stoichiometry, and suggest that FlhB is important not only for the regulation of substrate export but also for the opening of the export gate, adding to the complexity [112,202]. A cryo-EM and cryo-ET study of the *Salmonella* non-flagellar T3SS showed that thinning of the membrane around the export apparatus allows the export gate to span the membrane by docking the high-resolution FliPQR structure [210]. The accumulation of information about the export apparatus points towards a complex highly conserved in sequence, structure, assembly, and function, although the molecular mechanism underlying protein secretion remains poorly understood [193].

## 3. Specific Examples of Adaptation within the Bacterial Flagellum

**Figure 2:**
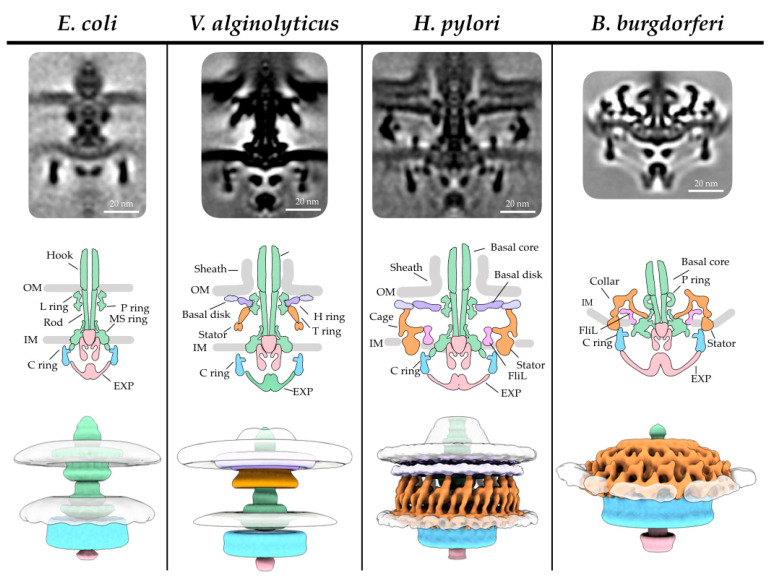
Intact flagellar motor structures reveal dramatic differences among species. Depicted for each species, from top to bottom, are the deposited class average of the motor, a cartoon model drawn from the class average, and a 3D reconstruction of the map. *E. coli* possess the simplest motor, resulting in a functional flagellum (EMDB 5311). *Vibrio spp.* have evolved additional rings that increase rotational speed. *H. pylori* (EMDB 8459), representing ε-proteobacteria, and *B. burgdorferi* (EMDB 0534), representing spirochetes, separately evolved structures that stabilize stators and increase rotor diameter, leading to greater torque generation.

Since the first intact flagellar motor was visualized in *Treponema primitia*, a spirochete with periplasmic flagella, by cryo-ET [127,179], a thorough investigation of 11 bacterial species using cryo-ET by Chen et al. highlighted the vast differences among flagellar motors, leading to new insights into bacterial evolution [113]. The ‘generic’ model created by Chen et al., by averaging motors from 11 different species, suggests that the hook, rod, and L, P, and MS rings are highly conserved morphologically. The motors in *Salmonella* and *E. coli* are the best-known examples of the generic model (Figure 2). However, the flagellar motors in other species have evolved unique structural features, presumably to adapt to different environments [113]. In this section, we highlight evolutionary differences by specifically examining three subsets of bacteria: marine *Vibrio*, ε- proteobacteria, and spirochetes.

### 3.1. Vibrio Flagella Have Additional Rings in the Periplasm for Greater Torque Generation

*Vibrio* species are marine bacteria that can cause gastroenteritis in humans via the consumption of contaminated water or seafood or via wound infections from swimming, with the well-known pathogen in this species being *Vibrio cholera*. The *Vibrio* single, polar, and sheathed flagellum has been studied in great detail biochemically (for example [211], for review of sheathed flagellum see [212]). Cryo-ET with STA revealed predominantly sheathed and, to a lesser extent, unsheathed flagella in wild-type *V. alginolyticus* (Figure 2). This allowed for the visualization of the membrane sheath and a novel O-ring structure [180]. A *V. alginolyticus flhG* mutant that assembles multiple polar flagella [213] was used to gain resolution due to more particles per cell pole, and as expected, the sheathed flagellum appears very different from the unsheathed flagella of *V. alginolyticus* and *E. coli*. The diameter at the base of the flagellum was larger due to the membranous sheath, and the loss of the outer membrane–L ring fusion led to more mobility of the basal body. Additional density, named the O ring, was observed outside of the outer membrane, creating a 90° kink in the outer membrane to form the sheath [180].

The *Vibrio spp.* motors also differ from *E. coli* and *Salmonella*, with the identification of the H (FlgT) and T (MotX and MotY) rings believed to have evolved to help the rotor spin faster, and stators that use Na^+^ ion pumps in lieu of the more common H^+^ ion pump composed of PomA/B [80,214,215]. The T ring was first identified via negative stain EM, whereby the Homma group showed that MotX and MotY form additional density associated with the LP rings and are required for PomA/B localization to the motor [214]. The H ring was later identified as FlgT and located above the T ring via negative stain EM [215]. A *V. fischeri ΔmotB* mutant showed that the stator interacts with the T ring, allowing for the wider incorporation of the stator relative to *Salmonella* and thus increasing the torque generation [80,83,121]. Further use of the *V. alginolyticus flhG* mutation strain in the presence of *ΔmotY* or *∆motX* suggests that the majority of the T ring is composed of MotY, as the *ΔmotY* mutation resulted in the loss of the T ring density, and the *ΔmotX* motors appeared relatively unchanged at low resolution. Importantly, the *V. alginolyticus* map revealed 13-fold symmetry of MotY, corresponding to the 13-fold symmetry of MotB in *V. ficsheri* [180]. Cryo-ET of *V. ficheri ΔflgP* [121] and *V. alginolyticus ΔflgO* and *ΔflgT* [216] mutants suggests that FlgT, FlgO, and FlgP create the proximal, medial, and distal regions of the H ring, respectively. In the *V. ficheri ΔflgP*, the stators did not assemble, and in the *V. alginolyticus ΔflgT* the flagella were periplasmic. Taken together, these results suggest that the H and T rings, unique to Na^+^ ion pump flagella, are required for proper flagellar assembly, stator association, and outer membrane penetration.

### 3.2. The ε-Proteobacteria Flagellum Cage Traps Additional Stators

*H. pylori* is a well-known gastrointestinal pathogen that can cause stomach ulcers and cancer. *H. pylori* cells possess unipolar, sheathed flagella which allow the microbe to swim through the stomach mucosal lining and are essential for host infection. The function of the sheath still remains unknown. One possibility is that it protects the filament from the low pH of the stomach. Cryo-ET of the *H. pylori* motor revealed a very large motor ~86 nm in diameter and ~81 nm in height (Figure 2) [57]. The motor consists of the basal body core structures along with a novel periplasmic “cage-like” structure. The cage structure had 18-fold symmetry, with the densities below occupied by the stators [57]. This scaffold likely evolved to secure the 18 stators for the high torque generation needed to swim though the viscous environment of the human stomach mucous [57]. *E. coli* require only 11 stators in their flagella, as identified by total internal reflection fluorescence microscopy (TIRF) [171,174]. Cryo-ET revealed similar stator scaffolds in *C. jejuni* [121] (a gut pathogen that causes food poisoning) and *Wolinella succinogenes* [114] (a cattle rumen commensal) motors, albeit with 17-fold symmetry, suggesting that these microbes possess 17 stators. In *C. jejuni*, deletion mutants *ΔflgP*, *ΔflgQ, ΔpflA*, *and ΔpflB*, were analyzed by cryo-ET to address questions of motor assembly and the composition of the basal and medial disks. It was determined that FlgP creates the basal disk, FlgQ and PflA create the medial disk, and PflB creates the proximal disk [121]. There is a notable difference in *C. jejuni*, where the medial ring is parallel to the proximal ring and basal disk, contrasting with the perpendicular medial ring in *H. pylori* and *W. succinogenes* [57,114]. These structural difference most likely arise due to the FlgQ sequence diversity [114]. Chaban et al. postulate that the energy demand for such a continuously high stator load may be offset by the nutrient-rich habitat, as all three species are part of the gut flora in animals.

### 3.3. The Periplasmic Flagella of Spirochetes Uses a Collar to Stabilize Stators

Spirochetes are a unique family of bacteria with distinct morphology and motility. Some of them are known to cause diseases such as leptospirosis (*Leptospira interrogans*), syphilis (*Treponema pallidum*), and Lyme disease (*B. burgdorferi)*. The flagella of spirochetes are unique due to the placement of the filament in the periplasmic space; this location has implications for the unique motility, host infection, and cell morphology of spirochetes [217] (for review [9]). From the first visualized in situ structures of the periplasmic flagellar motors in *T. primitia* [127] and *B. burgdorferi* [117,204], it has been readily apparent that the periplasmic flagella have a larger C ring, stator ring, and MS ring than those of *Salmonella* external flagella [12,218]. A spirochete-specific structure, also known as the collar, was identified [127]. The collar structure is approximately 71 nm in diameter and 24 nm in height, meaning the assembly is larger than the C ring [204] (Figure 2). 

The composition of the collar has recently been studied using *B. burgdorferi* as the model system [9,217]. To begin assigning *B. burgdorferi* proteins to the collar structure, all known flagellar proteins in *B. burgdorferi* were compared to those of externally flagellated genomes, and (BB0286) FlbB was identified as a potential hit. The *ΔflbB* mutant cells are rod-shaped and non-motile. Visualization of the *ΔflbB* motors by cryo-ET revealed that the collar did not assemble [219]. Furthermore, when *ΔflbB* was complemented, *flbB* fused with green fluorescent protein (GFP) extra densities near the MS ring were resolved, suggesting that FlbB constitutes the base of the collar and that other proteins must be involved in collar formation [219]. To further identify collar proteins, the *T. pallidum* protein–protein interaction map was used to identify homologs with FlbB and interactors [220]. The protein of unknown function (BB0236) was identified and characterized via molecular and cryo-ET experiments. BB0236 was determined to directly interact with FlbB in pull-down assays. Like the FlbB deletion mutant, *Δbb0236* resulted in non-motile, rod-shaped bacteria. Cryo-ET showed that BB0236 is necessary for collar formation as well as for FliL and stator assembly, suggesting that BB0236 is a chaperone protein that aids in the formation of the collar, and that the collar provides support for the assembly of the stator and FliL [221]. The most recently identified collar protein was determined by a blast search of the peptidoglycan binding loop of MotB. The gene product of *bb0326* was renamed FlcA. The *ΔflcA* mutant cells exhibited motility and morphology defects. Interestingly, cryo-ET demonstrated that the collar was assembled minus a region at the periphery, where FlcA resides. Density for FliL and FlbB was observed, suggesting that FlcA subsequently binds the collar. The stator was absent from the collar. FlcA was shown to interact with the stator protein MotB and the collar proteins FlbB and FliL, but not with BB0236 [222]. While the story of the spirochetal collar is still unfolding, cryo-ET combined with genetics has elegantly identified three proteins involved in collar assembly and shown the importance of the collar both for stabilization of the stator, by directly binding to MotB and the PG layer, and as a foundation for the stator assembly.

## 4. Conclusions and Perspectives

**Figure 3:**
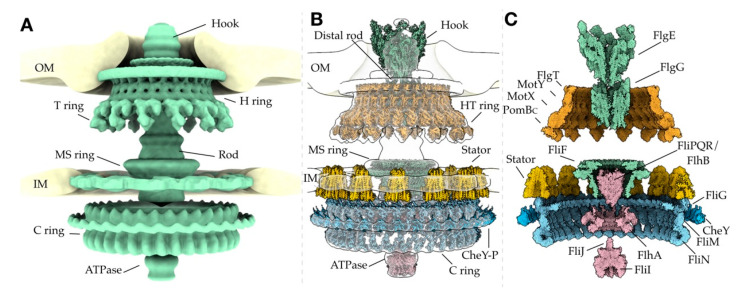
High-resolution cryo-EM and X-ray models placed in cryo-ET maps provide a basis for understanding flagellar assembly and function. (**A**). An assembled cryo-ET map of *z* motor trapped in the CW rotation (EMDB 3155, 21837, and [143]), depicting the general shape of the molecular components that assemble into the intact motor. (**B**). High-resolution cryo-EM and X-ray structures of the flagellar components are placed in the cryo-ET map (white). The motor is sliced in half to show the inner and outer structures. (**C**). Available high-resolution structures are shown in full. The models used for this reconstruction are: FlgE (PDB 6KFK), FlgG (PDB 6JZR), FlgT (PDB 3W1E), MotY (PDB 2ZF8), MotX (theoretical [181]), PomB_C_ (PDB 3WPW), FliF (PDB 6SD5), FliPQR–FlhB (PDB 6S3L), FliG (PDB 3HJL and 4FHR), CheY (PDB 1F4V), FliM (PDB 4FHR and 4YXB), FliN (PDB 4YXB and 1YAB), FlhA (PDB 6CH1), FliI (PDB 2DPY), FliJ (PDB 3AJW), and stator (PDB 6YKM).

Bacterial flagella have evolved as highly versatile nanomachines that enable bacteria to navigate and survive diverse environments such as the mucous of the mammalian gut. Over the last decade, cryo-ET has enabled direct visualization of conservation and adaptation of the bacterial flagellum to niche environments. Cryo-EM and X-ray crystallography have led to near-atomic views of purified flagellar proteins and subcomplexes, such as the MS ring, C ring, and stator complexes. By combining these techniques, it is becoming feasible to establish nearly complete models of the flagellar motor, such as the one shown in Figure 3. High-resolution views of the intact flagellar motor not only significantly enhance our understanding of flagellar structure and assembly but also provide the basis to address fundamental questions about bacterial flagella: How does proton motive force drive the flagellar assembly and rotation? How does the flagellum switch its rotational direction? And how has the flagellum evolved as a highly diverse nanomachine?

## Figures and Tables

**Table 1:** **Crystal structures of flagellar proteins.** A list of the flagellar protein structures deposited in the PDB.

Protein(s)	Species	PDB ID	Refs
**Axial**			
Flagellin FliC	*Bacillus cereus*	5Z7Q	[29]
	*Salmonella typhimurium*	1IO1	[30]
	*Sphingaomonas sp*	2ZBI, 3K8V, 3K8W	[31]
	*Burkholderia psuedomallei*	4CFI	[32]
	*Pseudomonas aeruginosa*	4NX9	[33]
FliS	*Aquifex aeolicus*	1ORY, 1ORJ	[34]
	*Bacillus cereus*	5XEF	[35]
	*Helicobacter pylori*	3IQC	[36]
FliT	*Salmonella typhimurium*	5GNA	
	*Yersinia enterocolitica*	3NKZ	
FljB	*Salmonella typhimurium*	6RGV	[37]
FcpA	*Leptospira biflexa*	6NQY	[38]
FcpB	*Leptospira interrogans*	6NQZ	[38]
Flagellin–FliS	*Bacillus subtilis*	5MAW, 6GOW	[39]
FliC–FliS fusion	*Aquifex aeolicus*	4IWB	[40]
FlgD	*Helicobacter pylori*	4ZZF, 4ZZK, 5K5Y	[41,42]
	*Salmonella typhimurium*	6IEE, 6IEF	
FlgE	*Campylobacter jejuni*	5AZ4	[43]
	*Caulobacter crescentus*	5AY6	[43]
	*Helicobacter pylori*	5NPY	[44]
	*Salmonella typhimurium*	1WLG	[45]
	*Treponema denticola*	6NDT, 6NDW, 6NDV, 6NDX	[46]
FlgK	*Campylobacter jejuni*	5XBJ	[47]
FlgL	*Bacillus cereus*	5ZIY	[48]
	*Xanthomonas campestris*	5ZIZ, 5ZJ0	[48]
	*Legionella pneumophila*	5YTI	
FliD (HAP2)	*Pseudomonas aeruginosa*	5FHY	[49]
	*Helicobacter pylori*	6IWY	[50]
FlgG	*Salmonella typhimurium*	6JF2	[51]
FlgJ	*Salmonella typhimurium*	5DN4, 5DN5	[52]
**Basal Body**			
FlgA	*Salmonella typhimurium*	3VKI, 3VJP, 3TEE	[53]
FliF–FliG	*Helicobater pylori*	5WUJ	[54]
FliF_c_–FliG_N_	*Thermotaoga maritima*	5TDY	[55]
FliG	*Aquifex aeolicus*	3HJL	[56]
	*Helicobacter Pylori*	3USY, 3USW	[57]
	*Thermotoga maritima*	1LKV, 1QC7, 3AJC	[58,59,60]
FliM	*Helicobacter pylori*	4GC8	[61]
	*Thermotoga maritima*	2HP7	[62]
	*Helicobacter pylori*	5XRW	[63]
FliN	*Thermotaoga maritima*	1YAB, 1O6A	[64]
FliY	*Thermotoga maritima*	4HYN	[65]
FliG–FliM	*Helicobacter pylori*	4FQ0	[61]
	*Thermotaoga maritima*	3SOH, 4FHR, 4QRM	[66,67,68]
FliM–FliN	*Salmonella typhimurium*	4XYB	[69]
FliM–FliN–FliH	*Salmonella typhimurium*	4XYC	[70]
FliM–SpeE	*Helicobacter pylori*	5X0Z	[71]
CheY	*Escherichia coli*	1U8T, 1ZDM, 2B1J, 2ID7, 2ID9, 2IDM, 6TG7	[72,73] [74]
	*Thermotoga maritima*	4IGA	[75]
CheY3	*Vibrio cholerae*	3TO5, 4H60, 4HNQ, 4JP1, 4LX8	[76]
CheY4	*Vibrio cholerae*	4HNR, 4HNS	[76]
CheY–FliM	*Escherichia coli*	1F4V	[72]
FlhG	*Geobacillus thermodenitrificans*	4RZ2, 4RZ3	[77]
MotB	*Salmonella typhimurium*	5Y3Z, 5Y40, 2ZVY, 2ZVZ, 2ZOV	[78,79]
PomB_c_	*Vibrio alginolyticus*	3WPW, 3WPX	[80]
MotY	*Vibrio alginolyticus*	2ZF8	[81]
FliL	*Vibrio alginolyticus*	6AHQ, 6AHP	[82]
FlgT	*Vibrio alginolyticus*	3W1E	[83]
**Export Apparatus**			
FlhA	*Bacillus subtilis*	3MIX	[84]
	*Salmonella typhimurium*	6CH1, 6AI0, 6AI1, 6AI2, 6AI3	[85,86]
FlhA FliT–FliD complex	*Salmonella typhimurium*	6CH2	[85]
FlhA FliS–FliC complex	*Salmonella typhimurium*	6CH3	[85]
FlhB	*Aquifex aeolicus*	3B1S	[87]
	*Salmonella typhimurium*	3B0Z	[87]
FlhF	*Bacillus subtilis*	2PX0, 2PX3	[88]
FliI	*Salmonella typhimurium*	2DPY	[89]
FliJ	*Salmonella typhimurium*	3AJW	[90]
FlgN	*Pseudomonas aeruginosa*	2FUP	
	*Salmonella typhimurium*	5B3D	[91]
FliH–FliI	*Salmonella typhimurium*	5B0O	[92]

**Table 2:** **Cryo-EM structures for flagellar subcomplexes**. A list of the cryo-EM maps and models deposited in the EMDB and PDB.

Protein(s)	Species	PDB ID	EMDB ID	Refs
**Axial**				
Flagellin	*Campylobacter jejuni*		5007	[93]
	*Salmonella typhimurium*	1UCU, 3A5X	1641	[94,95]
	*Bacillus subtilis*	5WJT, 5WJU, 5WJV, 5WJW, 5WJX, 5WJY, 5WJZ	8447, 8848, 8849, 8850, 8851, 8852, 8853	[96]
	*Pseudomonas aeruginosa*	5WK5, 5WK6	8855, 8856	[96]
	*Leptospira biflexa*	6PWB	20504	[38]
	*Salmonella typhimurium*	6JY0	9896	[97]
	*Kurthia spp.*	6T17	10362	[98]
FlgE	*Helicobacter pylori*	5JXL	8179	[99]
	*Caulobacter crescentus*	2BGY	1132	[100]
	*Salmonella typhimurium*	2BGZ, 3A69, 6JZT, 6KFK, 6K3I	1132, 1647, 9974, 9909	[100,101] [51,102,103]
	*Salmonella enterica*	6K9Q	9952	[104]
FliD (HAP2)	*Escherichia coli*		1873	[105]
FlgG	*Salmonella typhimurium*	6JZR	6683	[51]
**Basal Body**				
	*Salmonella typhimurium*		1887	[106]
FliF	*Salmonella typhimurium*	6SCN, 6SD1, 6SD2, 6SD3, 6SD4, 6SD5, 6TRE	10143, 10145, 10146, 10147, 10148, 10149, 10560, 6715	[107,108]
FliF–FliG	*Salmonella typhimurium*		6716	[108]
MotA	*Aquifex aeolicus*		3417	[109]
MotA/B	*Campylobacter jejuni*	6YKM, 6YKP, 6YKR	10828, 10829, 10830	[110]
	*Clostridium sporogenes*	6YSF	10895, 10897	[111]
	*Bacillus subtilis*	6YSL	10899	[111]
PomA/PomB	*Vibrio mimicus*		10901	[111]
**Export Apparatus**				
FliPQR	*Salmonella typhimurium*	6R69, 6F2D	4733, 4173	[107]
	*Vibrio mimicus*	6S3S	10096	[112]
	*Pseudomonas savastanoi*	6S3R	10095	[112]
FliPQR–FlhB	*Vibrio mimicus*	6S3L	10093	[112]
SctRST	*Salmonella typhimurium*	6R6B	4734	[107]

**Table 3:** *In situ* flagellar motors visualized by cryo-ET. A list containing the cryo-ET maps of flagellar motors deposited in the EMDB. Note that not all cryo-ET maps are deposited.

Species	EMDB ID	Refs
*Acetonema longum*	5297	[113]
*Arcobacter butzleri*	3910	[114]
*Borrelia burgdorferi*	0525, 0534, 0536, 0537, 0538, 1644, 5298, 5627, 5628, 5629, 5630, 5631, 5632, 5633, 6088, 6089, 6090, 6091, 6092, 6093, 6094, 6095, 6096, 6097, 6098, 9123, 21885, 21884, 21886	[113,115,116,117,118,119,120]
*Bdellovibrio bacteriovorus*	3911	[114]
*Campylbacter jejuni*	3150, 3157, 3158, 3159, 3160, 3161, 5300, 10341, 10342, 10343, 10345, 10454, 10455, 10456, 10457	[113,121,122]
*Caulobacter crescentus*	5312, 10943, 10945, 10949, 10950, 10955, 10956, 10957	[113,123]
*Escherichia coli*	5311	[113]
*Helicobacter pylori*	8459	[57]
*Heliobacter Hepaticus*	5299	[113]
*Hylemonella gracilis*	5309	[113]
*Hyphomonas neptunium*	5313	[113]
*Legionella pneumophila*	0464	[124]
*Leptospira biflexa*	20503, 20504	[38]
*Leptospira interrogans*	5912, 5913, 5914	[6]
*Plesiomonas shigelloides*	4569, 10057	[125]
*Pseudomonas aeruginosa*	0465	[124]
*Salmonella enterica*	2520, 2521, 3154, 3813, 5310	[113,121,126]
*Shewanella oneidensis*	0467	[124]
*Treponema primitia*	1235	[127]
*Vibrio cholerae*	5308	[113]
*Vibrio fischeri*	3155, 3156, 3162	[121]
*Vibrio alginolyticus*	21819, 21837	[128]
*Wolinella succinogenes*	3912	[114]
